# Supplementary material for: A Novel Nanomaterial-Based Approach for the Cryopreservation of Individual Sperm Cells Using Addressable Nanoliter Containers
Source: Nanomaterials (Basel). 2025 Jan 21;15(3):149. doi: 10.3390/nano15030149 (PMC11821078; doi:10.3390/nano15030149)
Supplement: Supplementary file 1 [file nanomaterials-15-00149-s001.zip › nanomaterials-3389593-supplementary.pdf]

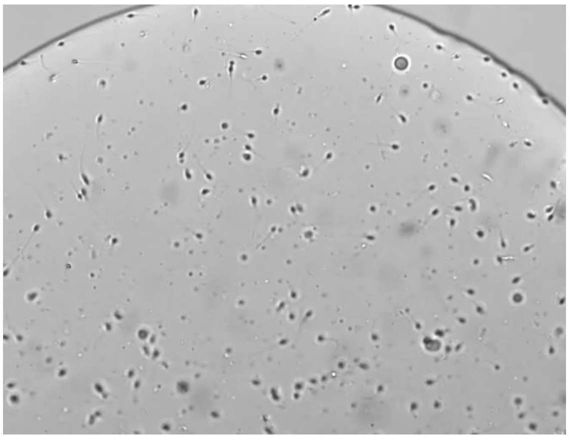

Video S1. Video clip taken under microscope. Link to video: https://drive.google.com/file/d/1nEZvK2e_r4TK1HwEmxRokYbpQM8b10x1/view?usp=sharing (accessed on 10 December 2024).

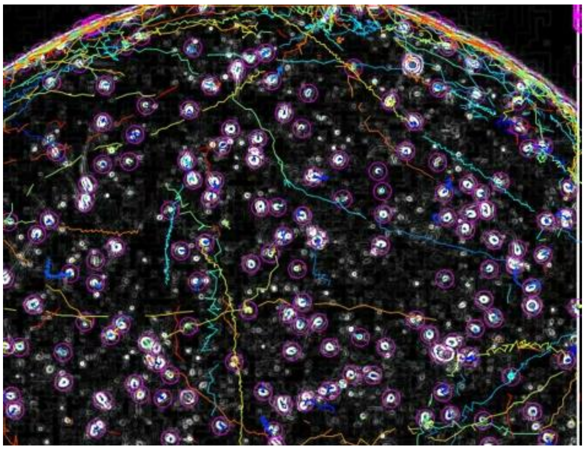

Video S2. Software output process. Link to video: https://drive.google.com/file/d/1Zc_ZBaqSy9PBEFtj0WzOIt6xh_j9PyYR/view?usp=sharing (accessed on 10 December 2024).

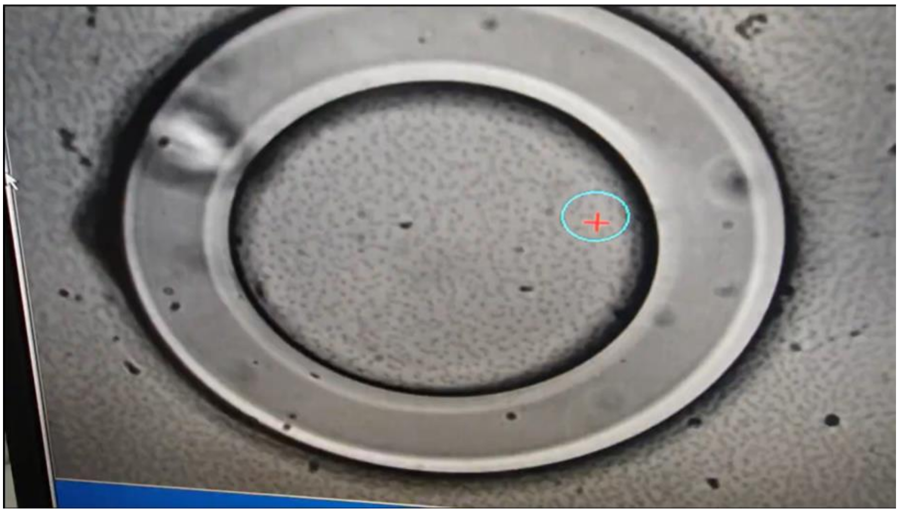

Video S3. Link to Video 3. Sperm cell after thawing (https://drive.google.com/file/d/1gBYReq3czlpY97LPZNtDQ7vQ_PVtWFyh/view?usp=sharing (accessed on 10 december 2024)).
